# Translocating *Mycobacterium ulcerans*: An experimental model

**DOI:** 10.1371/journal.pone.0230544

**Published:** 2020-12-30

**Authors:** Nassim Hammoudi, Mustapha Fellag, Muriel Militello, Amar Bouam, Michel Drancourt

**Affiliations:** 1 Aix-Marseille Univ., IRD, MEPHI, IHU Méditerranée Infection, Marseille, France; 2 IHU Méditerranée Infection, Marseille, France; Rutgers Biomedical and Health Sciences, UNITED STATES

## Abstract

*Mycobacterium ulcerans* is a non-tuberculous environmental mycobacterium responsible for extensive cutaneous and subcutaneous ulcers in mammals, known as Buruli ulcer in humans. *M*. *ulcerans* has seldom been detected in the faeces of mammals and has not been detected in human faeces. Nevertheless, the detection and isolation of *M*. *ulcerans* in animal faeces does not fit with the current epidemiological schemes for the disease. Here, using an experimental model in which rats were fed with 10^9^ colony-forming units of *M*. *ulceran*s, we detected *M*. *ulcerans* DNA in the faeces of challenged rats for two weeks and along their digestive tract for 10 days. *M*. *ulcerans* DNA was further detected in the lymphatic system including in the cervical and axillary lymph nodes and the spleen, but not in any other tissue including healthy and broken skin, 10 days post-challenge. These observations indicate that in some herbivorous mammals, *M*. *ulcerans* contamination by the digestive route may precede translocation and limited contamination of the lymphatic tissues without systemic infection. These herbivorous mammals may be sources of *M*. *ulcerans* for exposed populations but are unlikely to be reservoirs for the pathogen.

## Introduction

*Mycobacterium ulcerans* is an environmental, slow growing, non-tuberculous mycobacterium responsible for a progressively extending cutaneous and subcutaneous ulcer known as Buruli ulcer [1]. Buruli ulcer is a World Health Organization-notifiable neglected infection which has been notified by 34 countries over the last ten years [2]. It is a tropical infection mainly affecting rural populations in South America, West Africa, Australia, South China and Japan [3]. Although a genetic trait was recently described among seven individuals in a family, two of whom suffered from Buruli ulcer and carried a specific deletion on chromosome 8 [4], in addition to a previously reported deletion in the NRAMP-1 gene [5], Buruli ulcer is not a contagious infection but rather results from contact with *M*. *ulcerans*-contaminated environments [2]. Accordingly, it has been reported that *M*. *ulcerans* is cultivated from aquatic Hemiptera [6]. *M*. *ulcerans* DNA has also been detected in some animals including *Thryonomys swinderianus* (referred to in this paper as “agoutis”, the word commonly used in West Africa) [7, 8], rabbits and rats, which are all rodent mammals [9]. Moreover, two isolates of *M*. *ulcerans* have been reported in possum faeces collected in the region of Melbourne, Australia although characterisation and a repository reference were not provided [10]. These observations led to the suggestion that possums and *Pseudocheirus peregrinus* may play a role in the epidemiology of Buruli ulcer in endemic Australian regions [11].

Furthermore, we recently reported the isolation of *M*. *ulcerans* from *T*. *swinderianus* faeces collected in the vicinity of the Kossou Dam, Côte d'Ivoire, although we were unable to sub-culture this isolate [8]. These observations suggested that *T*. *swinderianus*, an herbivorous mammal rodent, may contaminate its digestive tract by the oral route after eating *M*. *ulcerans*-contaminated food; possibly acting as a secondary source of infection for populations, as it is hunted for bushmeat and eaten after unprotected, manual evisceration [7] Likewise, it has been suggested that the small terrestrial mammals *Mastomys natalensis* may play a potential role in the natural history of *M*. *ulcerans* [12]. In addition, we recently observed the PCR-based detection of the ketoreductase B gene (KR-b) and the IS*2404* and IS*2606* insertion sequences in one tenth of spleen specimens collected from *T*. *swinderianus* in the area of Yamoussoukro, Côte d’Ivoire, and in spleen of common ringtail possums in some areas of Victoria that are endemic for *M*. *ulcerans* disease [7–10].

Taken together, these observations point to the medical interest of understanding the mode of contamination and pathology of *M*. *ulcerans* in *T*. *swinderianus*. We therefore developed an experimental model of the oral contamination route in rats, asking four questions relative to the survival and excretion of the pathogen in the digestive tract, its systemic dissemination, and its ability to translocate and to further infect previous aseptic skin lesions.

## Materials and methods

### Ethics statement

The experimental protocol, registered by the French Ministry of Higher Education and Research under reference number 2018081011226001, was approved by the Institutional Animal Care and Use Committee of Aix-Marseille University “C2EA- 14”, France after a revised version was approved 17 weeks after the initial submission. All animal handling was carried out in accordance with the rules of French Decree N° 2013–118, 7 February 2013 and the experimental procedures on rats were carried out in accordance with European law and in agreement with Animal: Reporting In Vivo Experiments (ARRIVE Guidelines http://www.nc3rs.org.uk); both prohibiting any cruel experimentation. We used Long-Evans rats (Charles River Laboratories, L'Arbresle, Lyon, France). The animals were housed in protected environmental area, in individual transparent cages (one rat per cage) in individually ventilated Allentown Technologies caging systems (Allentown, Pennsylvania, USA) with free access to a standard diet including dehydrated rodent feed pellets and sterile water until the experiment. Efforts were made to minimise the number of animals and to limit their stress by enriching their environment with litter and cardboard tunnels. The rats were observed daily for any signs of distress or adverse events. To minimise the animals’ suffering, all invasive procedures were performed under full general anaesthesia. The animals were sacrificed by an injection of a lethal dose of Pentobarbital, preceded by full general anaesthesia. All experiments were performed in a biosafety level 3 laboratory at the University Hospital Institute (IHU), Marseille, France.

### *M*. *ulcerans* inoculum

*M*. *ulcerans* strain CU 001, a clinical isolate from Ghana [13] was cultured on Middlebrook 7H10 supplemented by OADC over a six-week incubation period at 30°C. Colonies were suspended on a sterile phosphate buffered saline (PBS) tube and the bacteria were vigorously vortexed for ten minutes using 3-mm sterile glass beads (Sigma-Aldrich, Saint-Quentin- Fallavier, France) and passed three times through a 29 Gauge in order to eliminate bacterial aggregates. The mycobacterial suspension was then calibrated at optic density of 5 McFarland (equivalent to 10^9^ colony-forming units (CFU)/mL.

### Rat challenge protocol

Animal experiments were performed on 16 rats (eight males and eight females) aged eight weeks (Charles River Laboratories) and weighing between 220g and 250g. These animals, which were obtained with complete health reports, were found to be healthy and free of infection and were housed under conditions free of specific pathogens. Each rat was placed into an individual plastic cage with free access to water and food. In order to test the hypothesis of internal contamination and induction of Buruli ulcer on pre-existing skin lesions, a skin lesion was artificially created. For this purpose, rats received a full general anaesthesia with an intraperitoneal injection of a mixture of Ketamine (80 mg/kg) and Xylazine (10 mg/kg). One gram of sterile glass powder mixed with 300 μL of sterile PBS was injected under the skin on the right shoulder and 300 μL of PBS under the skin on the left shoulder as a negative control. Digestive inoculation was performed after the shoulder skin lesions resolved (i.e., seven days after skin lesions were made). Briefly, the rats were manually restrained and sterile, single-use 1-mL syringes were used to administer the *M*. *ulcerans* suspensions directly into the rats’ mouths, respecting the swallowing cycle in order to ensure that rats swallowed the entire administered suspension: 300 μL of sterile PBS were administered to four rats (two males and two females) forming the negative control group and 300 μL of mycobacterial suspension at 10^9^ CFU/mL was administered to 12 challenged rats (six females and six males).

### Animal follow-up and sample collection

The rats’ behaviour was observed daily until the day of euthanasia. In the post-infectious period, animals were closely observed on a daily basis for any abnormal behaviour including swelling/bleeding at the injection site, ruffled coats, hunched posture, and any signs of pain or distress. Faeces were collected directly from the exit of the rectum on the first day of challenge and then every two days until 20 days after the challenge. Ten days after the challenge, rats in the first group of six infected rats (three males and three females) and two controls (one male and one female) were sacrificed by intraperitoneal injection of a lethal dose of Pentobarbital (100–150 mg/kg) preceded by full general anaesthesia as previously described. The organs were carefully collected. A second group of six infected rats (three males and three females) and one male and one female control rats were monitored in the same way until 60 days after the challenge, then sacrificed by intraperitoneal injection of a lethal dose of Pentobarbital (100–150 mg/kg) preceded by full general anaesthesia as previously described and euthanised to collect a second set of organs.

### PCR detection of *M*. *ulcerans* DNA

All collected organs were stored in Eppendorf tubes at 4°C for one day. One piece of each organ was then transferred to another 1.5 mL-Eppendorf tube containing 500 μL of sterile PBS. The organs were vigorously crushed using a single use sterile piston and 200 μL of organ juice were put into a 1.5 mL-Eppendorf tube containing a mixture of 200 μL G2 lysis buffer, 20 μL proteinase K and a small quantity of glass powder. The tube underwent three cycles of FastPrep 24^TM^-5 (MP Biomedicals, Strasbourg, France) before being heated to 56°C for two hours. 200 μL of supernatant was then used to extract DNA using the EZ1 apparatus according to the manufacturer’s recommendations (Qiagen, GmbH, Germany). Extracted DNA was stored at 4°C. Detection of *M*. *ulcerans* DNA was performed by using real-time PCR (RT-PCR) and a CFX thermal cycler (BIO-Rad, Marnes-la-Coquette, France) using specific primers targeting the ketoreductase B gene (KR-b) and the IS*2404* and IS*2606* insertion sequences, as previously described [14]. The negative controls of our RT-PCR reactions were formed from the same reaction mix as our samples, switching only the 5μL of DNA for 5 μL of ultrapure™ DNase/RNase-Free Distilled Water (Invitrogen France). One negative control was placed after every five samples on a Light Cycler 480 multiwell plates 96-well plate (Roche).

### *M*. *ulcerans* culture

One piece of spleen collected from each rat was crushed in 500 μL of sterile PBS using sterile single-use piston and 2 X 150 μL of spleen juice was inoculated on two Middlebrook 7H10 agar plates incubated at 30°C for two months. In addition, the faeces collected at 3, 14- and 20-days post-challenge were incubated for ten minutes at room temperature in 2 mL NAOH (1M) and centrifuged at 3,500 g for ten minutes. The pellet was resuspended in 1 mL of 5% oxalic acid and incubated for ten minutes at room temperature before centrifugation at 3,500 g for a further ten minutes. The pellet was then resuspended in 2 mL home-made Trans MUl decontamination-preculture medium and incubated for five days at 30°C. The mixture was then vortexed 30 times and 200 μL was plated onto Trans MUg, a homemade Middlebrook-based medium supplemented with 10% oleic acid, bovine albumin, dextrose and catalase enrichment (OADC, Becton Dickinson), oxytetracycline (40 μg/mL), polymyxin E (80 μg/mL) and voriconazole (50 μg/mL) for three months at 30°C. Trans MUg medium was previously tested and shown to respect the viability of *M*. *ulcerans* (no published data).

## Results

### Rat clinical follow-up

During a 60-day post-challenge follow-up period, all rats in the control group and all rats in the challenged group remained apparently healthy and displayed no pathological clinical signs, no pain and no weight loss. In addition, the subcutaneous lesions induced by the glass powder displayed no pathological signs during the experiment.

### RT-PCR test results

In all RT-PCR assays, the negative controls remained negative. Ten days after the challenge, the RT-PCR detection of *M*. *ulcerans* DNA remained negative on the internal organs and faeces collected in control group rats whereas it was positive in some of the *M*. *ulcerans*-challenged rats. RT-PCR tests revealed a simultaneous detection of IS*2606* insertion sequence and the KR-B gene in the cervical lymph nodes, axillary lymph nodes, spleen and digestive tract in males and females (Table 1, S1 Table). *M*. *ulcerans* DNA was also detected in the faeces of challenged rats up to 15- and 17-days post-challenge, in males and females, respectively. At 60 days post-challenge, the RT-PCR detection of *M*. *ulcerans* DNA remained negative on the internal organs and in faeces collected in control and challenged groups (Table 1, S1 Table).

**Table 1:** Detection of *M*. *ulcerans* in the organs and faeces collected from rats challenged with the pathogen by the oral route.

		Male rats				Female rats			
		A	E	F	G	B	H	I	J
Rats sacrificed at 10 days post-challenegativeinegative	Left lesion	negative	negative	negative	negative	negative	negative	negative	negative
	Right lesion	negative	negative	negative	negative	negative	negative	negative	negative
	Cervical ganegativelion	negative	37.2–38.1	37.78–38.33	38.4–38.94	negative	37.01–37.73	37.67–38.11	negative
	Axillary ganegativelion	negative	37.24–38.72	38.67–39.11	37.35–38.15	negative	negative	negative	38.67–38.47
	Heart	negative	negative	negative	negative	negative	negative	negative	negative
	Lunegative	negative	negative	negative	negative	negative	negative	negative	negative
	Spleen	negative	37.5–38.79	negative	38.4–38.66	negative	37.24–38.29	38.92–38.56	negative
	Kidney	negative	negative	negative	negative	negative	negative	negative	negative
	Liver	negative	negative	negative	negative	negative	negative	negative	negative
	Stomach	negative	37.35–38.65	37.36–38.33	37.37–38.46	negative	37.4–38.52	37.27–38.70	37.35–38.89
	Small intestine	negative	38.1–39.21	38.2–38.78	38.3–38.55	negative	36.44–37.89	37.67–39	38.25–39.02
	Large intestine	negative	37.68–38.82	37.68–38.83	37.68–38.7	negative	37.01–37.79	38.26–38.29	37.84–38.69
	Secum	negative	37.26–3845	37.26–38.84	37.26–38.29	negative	37.42–39.01	negative	37.27–38.84
	Peyer plate	negative	38.74–38.95	38.74–39	38.75–38.65	negative	38.01–38.73	38.62–38.69	38.72–39.12
	Mesenteric nodes	negative	38.87–39.29	38.87–38.91	negative	negative	39.11–38.49	38.59–38.72	38.92–38.48
	Feces		**1—>10**	**1—>10**	**1—>10**		**1—>10**	**1—>10**	**1—>10**
		Male rats				Female rats			
		C	K	L	M	D	N	O	P
Rats sacrificed at 60 days post-challenegativeinegative	Left lesion	negative	negative	negative	negative	negative	negative	negative	negative
	Right lesion	negative	negative	negative	negative	negative	negative	negative	negative
	Cervical ganegativelion	negative	negative	negative	negative	negative	negative	negative	negative
	Axillary ganegativelion	negative	negative	negative	negative	negative	negative	negative	negative
	Heart	negative	negative	negative	negative	negative	negative	negative	negative
	Lunegative	negative	negative	negative	negative	negative	negative	negative	negative
	Spleen	negative	negative	negative	negative	negative	negative	negative	negative
	Kidney	negative	negative	negative	negative	negative	negative	negative	negative
	Liver	negative	negative	negative	negative	negative	negative	negative	negative
	Stomach	negative	negative	negative	negative	negative	negative	negative	negative
	Small intestine	negative	negative	negative	negative	negative	negative	negative	negative
	Large intestine	negative	negative	negative	negative	negative	negative	negative	negative
	Secum	negative	negative	negative	negative	negative	negative	negative	negative
	Peyer plate	negative	negative	negative	negative	negative	negative	negative	negative
	Mesenteric nodes	negative	negative	negative	negative	negative	negative	negative	negative
	Feces	negative	**1—>15**	**1—>14**	**1—>15**	negative	**1—>15**	**1—>17**	**1—>14**

Grey squares indicate the absence of detection, white squares indicate positive detection with the Ct-values of the KR-b gene—IS*2606*. “A-B-C-D” denotes negative control, non-challenged rats; “E-P” denotes challenged rats. The numbers in bold indicate the time (days) when *M*. *ulcerans* DNA was detected in the faeces.

### Culture results

No colonies of *M*. *ulcerans* were isolated from the faeces and spleen samples collected from the four negative-control group rats after three months of incubation. The same observations were reported after three months of cultivation of faeces and spleens of 12 *M*. *ulcerans*-infected rats.

## Discussion

We previously reported the molecular detection of *M*. *ulcerans* DNA in the digestive tracts and spleens of wild agoutis caught in Côte d'Ivoire, and its culture in one case [7, 8]. In this study, we developed an experimental model of gastrointestinal inoculation in rats to confirm that our field observations were the result of the probable gastrointestinal contamination of wild agoutis. We chose the rat model as it is the laboratory mammal closest to the agouti, sharing its general morphology (in terms of size and weight), an herbivorous rodent diet, and a body temperature of 37°C, which also makes it a relevant model of human infection [15].

The rat model showed that these rodents can be inoculated by *M*. *ulcerans* through the digestive tract, allowing us to answer our first question. Indeed, the negative control rats and the experimental negative controls that we introduced at all stages of our experiments remained negative in both the RT-PCR and the culture-based experiments carried out on the faeces and internal organs RT-PCR assays of uninfected rats. This allowed us to interpret the positive results we observed as authentic and not a mere result of laboratory contamination.

Our experimental results thus confirm that some wild rodents are digestively infectable with *M*. *ulcerans*, as has been previously observed for wild agoutis in Côte d'Ivoire [7, 8] and for possums in Australia [10]. Accordingly, our results reporting *M*. *ulcerans* DNA detected in faeces and in internal organs suggest that some wild rodents may participate in the natural cycle of the pathogen. This observation indicates that direct contact with the faeces of these wild rodents, when they are prepared as bushmeat for example, constitutes a circumstance of contamination of populations by live *M*. *ulcerans*, and potentially a source of Buruli ulcer.

In a second step, *M*. *ulcerans* was detected by RT-PCR but not cultured in the spleen and some lymph nodes of challenged rats but not in negative control rats. This observation indicates that *M*. *ulcerans* probably has the ability to penetrate the digestive mucosa and to be retained by the lymphatic system in which it is destroyed. This experimental result corroborates the fact that *M*. *ulcerans* has never been detected in tissue or organs at a distance from its inoculation point. Further, our observations suggest that mycolactones detected by liquid chromatography coupled with mass spectrometry in skin lesions [16] and in the blood of Buruli ulcer patients [17] may have no systemic cytotoxic activity. It should be noted that this translocation property is shared with mycobacteria of the *Mycobacterium tuberculosis* complex, in which experimental translocation has been shown for *Mycobacterium canettii* [18] and *M*. *tuberculosis* [18]. There are no experimental data or clinical observations to our knowledge of mycobacteria of the *Mycobacterium leprae* complex. Our results also indicate that *M*. *ulcerans* shares with the *M*. *tuberculosis* complex mycobacteria the property to infect the lymphatic tissue, albeit with an abortive form of the infection [19]. Accordingly, *M*. *ulcerans* was not cultured from any internal organ (linked to the unfavorable 37°C body temperature of the rat).

After translocation, the fate of *M*. *ulcerans* differs considerably from that of mycobacteria of the *M*. *tuberculosis* complex. The latter spread to the lungs and other highly vascularised organs [20], while *M*. *ulcerans* did not spread into any organs in the rat model.

Taken together, the observations here reported indicate that in some herbivorous mammals, *M*. *ulcerans* contamination by the digestive route may precede translocation and limited contamination of the lymphatic tissues without systemic infection. These herbivorous mammals may participate as sources of *M*. *ulcerans* for exposed populations but are not reservoirs for the pathogen.

## Supporting information

S1 Table (XLSX)
